# Ultrafast quantum computation in ultrastrongly coupled circuit QED systems

**DOI:** 10.1038/srep44251

**Published:** 2017-03-10

**Authors:** Yimin Wang, Chu Guo, Guo-Qiang Zhang, Gangcheng Wang, Chunfeng Wu

**Affiliations:** 1 College of Communications Engineering, PLA University of Science and Technology, Nanjing 210007, China; 2 Quantum Physics and Quantum Information Division, Beijing Computational Science Research Center, Beijing 100193, China; 3 Pillar of Engineering Product Development, Singapore University of Technology and Design, 8 Somapah Road, 487372, Singapore; 4 Center for Quantum Sciences and School of Physics, Northeast Normal University, Changchun 130024, China

## Abstract

The latest technological progress of achieving the ultrastrong-coupling regime in circuit quantum electrodynamics (QED) systems has greatly promoted the developments of quantum physics, where novel quantum optics phenomena and potential computational benefits have been predicted. Here, we propose a scheme to accelerate the nontrivial two-qubit phase gate in a circuit QED system, where superconducting flux qubits are ultrastrongly coupled to a transmission line resonator (TLR), and two more TLRs are coupled to the ultrastrongly-coupled system for assistant. The nontrivial unconventional geometric phase gate between the two flux qubits is achieved based on close-loop displacements of the three-mode intracavity fields. Moreover, as there are three resonators contributing to the phase accumulation, the requirement of the coupling strength to realize the two-qubit gate can be reduced. Further reduction in the coupling strength to achieve a specific controlled-phase gate can be realized by adding more auxiliary resonators to the ultrastrongly-coupled system through superconducting quantum interference devices. We also present a study of our scheme with realistic parameters considering imperfect controls and noisy environment. Our scheme possesses the merits of ultrafastness and noise-tolerance due to the advantages of geometric phases.

Quantum computing has attracted much attention due to its acknowledged potential in solving hard problems over its classical counterparts, such as prime factoring, database searching, and etc. Superconducting circuit systems are promising platforms for quantum computation and quantum simulation because of its exotic properties such as scalability, controllability, flexibility, and compatibility with micro-fabrication^1^. In addition to the well-known three kinds of superconducting qubits: charge qubit^2^, flux qubit^3^, and phase qubit^4^, newly-designed qubits, such as transmon qubit^5^,^6^, fluxonium^7^, Xmon qubit^8^,^9^ have been widely explored for quantum information science. Considerable progresses have been made in recent superconducting circuit experiments involving the observation of the dynamical Casimir effects^10^, the realization of adiabatic quantum gate operations^11^, the demonstration of digital quantum simulation^12^, and so on. Especially, the ultrastrong coupling (USC) regime of light-matter interaction has been reached when a flux qubit is galvanically connected to a coplanar waveguide resonator^13^,^14^, where the qubit-resonator coupling strength *g* is comparable to the resonator frequency *ω*_*r*_: 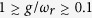
15. This coupling regime not only provides a demand to study the quantum Rabi model^16^,^17^ and interesting quantum optics phenomena^18^,^19^,^20^,^21^,^22^,^23^,^24^, but it also leads to fast quantum gate operations^25^,^26^,^27^ as well as holonomic quantum computations^28^. Specifically, a theoretical proposal to make ultrafast two-qubit quantum gates in circuit QED at the time scale of sub nanoseconds (0.1 ns) has been proposed in 2012^25^, but it requires controllable coupling ratios as large as 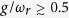. Although a deep strong coupling ratio of 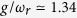 between a flux qubit and a LC oscillator has been achieved very recently with a large inductor^29^, quantum computation with very large coupling strength and multiple qubits remains technically challenging.

On the other hand, one of the major practical difficulties in building up a quantum computer is that quantum systems are inevitably influenced by the decoherence effect induced from the environment. Decoherence will collapse the state and render the quantum process invalid. It is thus essential to consider noise-tolerant proposals to implement quantum gates. One possible way is to resort to the geometric phase, which depends only on the solid angle enclosed by the parameter path^30^ and generally not on the dynamics of the evolution^31^. Geometric quantum computation has been experimentally implemented in various quantum systems such as thermal ensembles of molecules^32^, solid-state spins^33^, superconducting qubits^34^ and so on.

In this work, we explore an ultrastrongly coupled qubit-resonator system involving flux qubits galvanically connected to a transmission line resonator (TLR), which is linked to another two empty resonators by superconducting quantum interference devices. The evolution of the system may result in a nontrivial two-qubit geometric phase gate, which is achieved from the displacements of the three-mode intracavity fields. Since there are three resonators contributing to the phase accumulation, it is possible to largely relax the requirement of very large coupling strength needed to achieve the phase gate as compared to the gate proposal in ref. 25. Further reduction in the coupling strength to realize a certain quantum controlled-phase gate can be achieved by adding more auxiliary resonators to the ultrastrongly-coupled system. Our result makes an essential step forward for multi-resonator circuit QED and can be used to implement ultrafast quantum gates bearing noise-tolerant merits by resorting to the advantages of geometric phase.

## Results

### The superconducting circuit model

**Figure 1:**
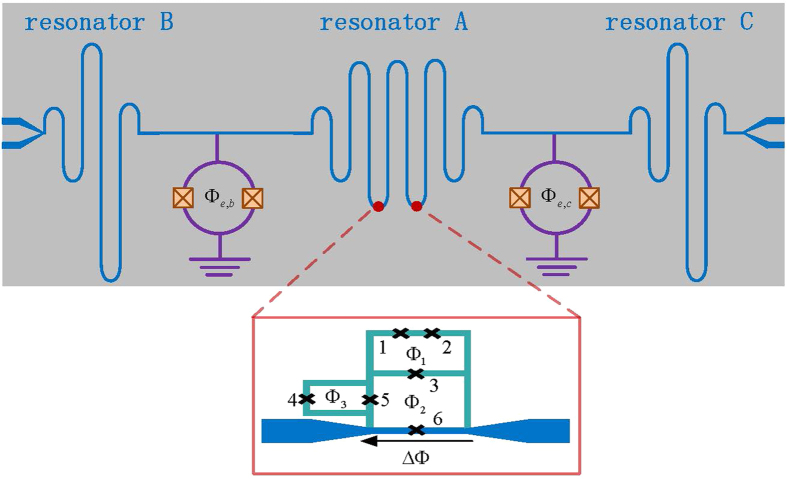
Schematic of a circuit-QED design for our proposal. Two flux qubits, each of which is composed of three Josephson junctions (1, 2, 3) in the upper loop threaded by an external flux Φ_1_, are galvanically connected to the center conductor of the middle transmission line resonator (TLR). Tunable qubit-resonator couplings are achieved by controlling the external fluxs Φ_2_, Φ_3_ in the additional loops. Another two transmission line resonators, which are coupled to the middle TLR through the corresponding SQUIDs, are used as ancillary systems to accelerate the quantum computation.

We propose the use of ancillary microwave resonators as a tool to further accelerate the protected quantum computation in the ultrastrong coupling regime. As schematically shown in Fig. 1, two empty TLRs are coupled to the two sides of the TLR through a superconducting quantum interference device (SQUID)^10^,^35^,^36^,^37^,^38^,^39^. The SQUID couplers can also be replaced by capacitor couplers^40^,^41^ or qubit couplers^42^,^43^. In the middle TLR, two identical flux qubits, each of which is composed of three Josephson junctions (JJ), are uniformly distributed and galvanically connected to the center conductor by means of the coupling junctions, i.e. JJ_6_. This qubit-circuit configuration allows for ultrastrong qubit-resonator coupling strength. Moreover, the magnetic flux Φ_2_ and Φ_3_ in the two additional loops provide its tunability and switchability^25^,^44^. When the inductance of the sixth junction is much smaller than the sum of inductances belonging to the loop threaded by the external flux Φ_2_, its presence can be approximately understood as a perturbation to the TLR. We further impose that the JJ_6_ operates in a linear response regime with 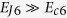, which results in a nonlinear resonator spectrum. We consider the case that the flux qubits are sitting in the degeneracy point of the resonator, where a set of eigenmodes become degenerate^27^,^45^. In this single-band approximation, the Hamiltonian of the resonator is of the form 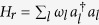 (setting 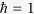). The Hamiltonian of the total setup becomes (the detailed derivations can be found in ref. 25)


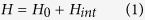



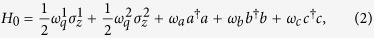



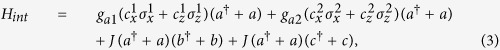


where 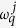 is the frequency of the *j*th qubit, 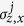 are the corresponding Pauli matrices, *ω*_*a,b,c*_ are the frequencies of the middle, left, right TRL resonators, *a*^†^(*b*^†^, *c*^†^) and *a(b, c*) are the creation and annihilation operators for the corresponding microwave resonators. The resonator-resonator coupling strength *J* can be controlled by the external magnetic flux Φ_*e,b*_ and Φ_*e,c*_ threading the SQUID.

The qubit-resonator coupling strength 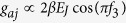 depends on the external magnetic flux Φ_3_ and the size *β* of the fourth and the fifth Josephson junction. The coefficients 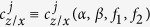, which satisfy the condition 
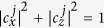
 for 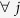, are the magnitudes of the longitudinal/transverse qubit-resonator coupling strengths. They can be controlled by the qubit junction size 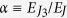, 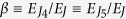 and the external fluxes Φ_2_ and Φ_3_. In particular, it might be possible to switch from transversal to longitudinal coupling by making 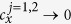, 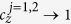
25,27. Thus the Hamiltonian of the total system is reduced to


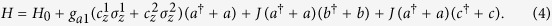


We next perform a unitary transformation on the system Hamiltonian with


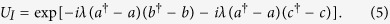


By performing rotating wave approximation respecting the condition of 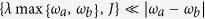, we arrive at the following effective Hamiltonian,


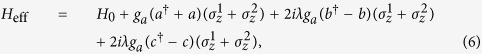


where the constant term has been omitted.

### Two-qubit controlled-phase gate

Here, we try to implement two-qubit quantum phase gate in the computational basis 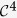 of 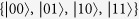 spanned by the two flux qubits. The Hamiltonian Eq. (6) can thus be expressed diagonally as 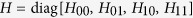 in the interaction picture, with the elements *H*_*ij*_ given by


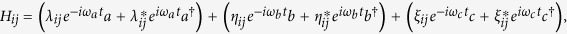


where 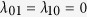, 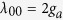, 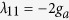, 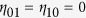, 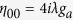, 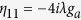, 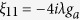, 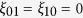, and 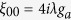. The evolution matrix *U(t*) is also in a diagonal form in 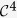, as according to the Hamiltonian in Eq. (6), the states will not evolve out of 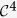. The corresponding elements *U*_*ij*_(*t*) are nothing but the displacement operators 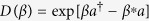 together with the accumulated phases^46^,


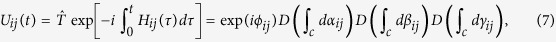


where 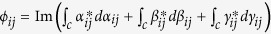, 
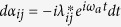
, 
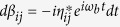
, and 
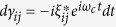
. The phase *ϕ*_*ij*_ accumulated by the state |*ij*〉 via the interaction between flux qubits and the resonators is the unconventional geometric phase^40^,^47^,^48^,^49^ and hence the phase *ϕ*_*ij*_ is robust against certain type of noise as it bears global geometric features.

We assume that the resonators are initially in vacuum state, and at any time *t* > 0, the states of the resonators evolve to coherent states with amplitudes *α*_*ij*_(*t*), *β*_*ij*_(*t*) and *γ*_*ij*_(*t*) depending on the logical computational basis state |*ij*〉. We find 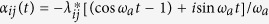, 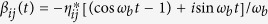 and 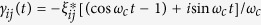. By choosing *ω*_*b*_ = *ω*_*c*_ = *ω*_*a*_/*m*, after a process operating at *T* = 2*π*/*ω*_*b*_, the phase *ϕ*_*ij*_ is found to be 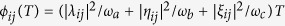. As such the total operation time is *T*, and at time *T* the time evolution matrix can be written as


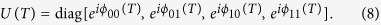


*U(T*) is thus a nontrivial entangled gate if and only if 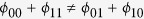
50. This condition can be fulfilled by adjusting the coupling strengths. In our scheme the phases acquired by the qubits to realize a certain phase gate are due to the close-loop evolution of the resonators. In particular, let us try to realize a two-qubit gate with *ϕ*_00_ = *ϕ*_11_ = *π*/2 and *ϕ*_01_ = *ϕ*_10_ = 0. In this case, the qubit-resonator normalized coupling strength needs to satisfy


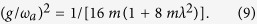


From Eq. (9), we see that the coupling strength required to construct a two-qubit gate is largely reduced as compared to the required coupling ratio of 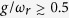 in ref. 25. By adjusting the displacement amplitude to be *λ* = 0.1, the ratio to be *m* = 2, and the qubit-resonator coupling strength to be *g*/*ω*_*a*_ ~ 0.16 as satisfying Eq. (9), we have a two-qubit gate with operation time of 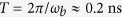 if the frequency of the middle resonator is *ω*_*a*_/2*π* = 10 GHz.

**Figure 2:**
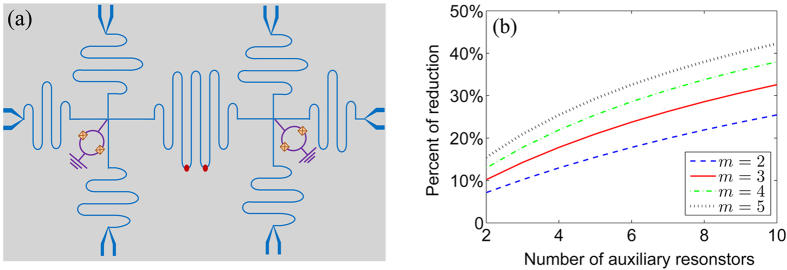
Multi-resonator case. (**a**) Six transmission line resonators from left and right sides are connected to the middle transmission line resonator through the corresponding SQUIDs. This scheme allows for large-reduction of the normalized coupling strength to achieve a specific phase gate. (**b**) The percentage of the reduction on the requirement of the normalized coupling strength for a specific phase gate in the multi-resonator case.

It is worthy to note that, to further reduce that requirement on the normalized coupling strength to achieve a specific phase gate, we can easily extend our model to the multiple resonators case for assistance, as schematically shown in Fig. 2(a). With N auxiliary resonators, the normalized coupling strength reads


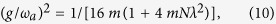


where *N* is number of the auxiliary resonators. With this general equation in Eq. (10), the percentage of the reduction in the normalized coupling strength for the multi-resonator case is shown in Fig. 2(b). Therefore, it is noteworthy that in addition to the operational robustness of the geometric phase gates, another advantage offered in our scheme by employing auxiliary resonators is the decrease of the coupling strength on demand to achieve a certain gate. This result is thus of applicable importance for quantum computation and quantum information processing. In practical study, a desirable quantum gate may require relatively large coupling strength, which is not possibly available based on current techniques. An alternative way to realise such a gate is to use the auxiliary resonators, in which we need smaller strong coupling strength.

**Figure 3:**
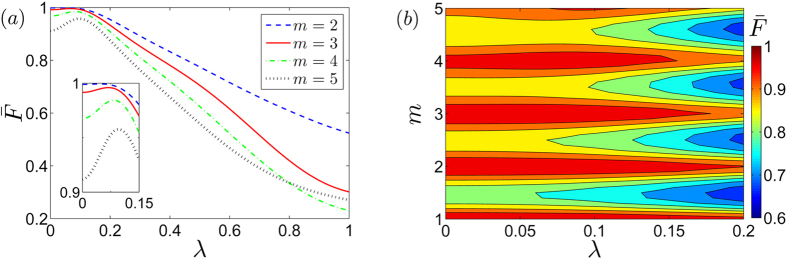
Gate fidelity plots. (**a**) Gate fidelity 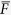 as a function of the displacement magnitude *λ* for different values of *m*. The inset is a zoom of the region for 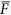 between 0.9 and 1. (**b**) Contour plots of the gate fidelity 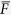 as a function of the displacement magnitude *λ* and the ratio *m*. In this simulation to achieve a nontrivial two-qubit phase gate *U(T*) with *ϕ*_00_ = *ϕ*_11_ = *π*/2 and *ϕ*_01_ = *ϕ*_10_ = 0, we choose the system parameters as cavity frequencies *ω*_*a*_/2*π* = 10 GHz, *ω*_*b*_ = *ω*_*c*_ = *ω*_*a*_/*m*, the total evolution *T* = 2*π*/*ω*_*b*_, and the resonator-resonator coupling strength *J* = 0.1|*ω*_*a*_ − *ω*_*b*_|.

We next explore the performance of the two-qubit quantum gate by resorting to multi-resonator circuit QED systems, and show the advantages offered by the auxiliary resonators. For a particular initial state of the system in the logical qubit basis


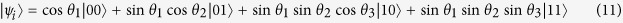


with 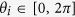 (*i* = 1, 2, 3), the state fidelity


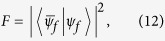


is defined between the expected final state 
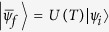
 and the state 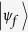 after the evolution of the scheme. Taking the maximally entangled state 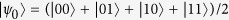 being the initial state as an example, we find that the error of the quantum phase gate due to the approximations involved in the derivation of the effective Hamiltonian is less than 0.01. To better explore the gate performance, we define the gate fidelity as the fidelity averaged over all the possible states,


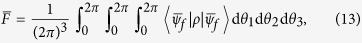


with *ρ* being the density matrix of the two logical qubits after evolution. In Fig. 3(a), we show the calculated gate fidelity 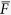 as a function of the displacement magnitude *λ* for different values of the ratio *m*. In Fig. 3(b), we show the optimal operating conditions to obtain the maximum gate fidelity. As shown in Fig. 3, to achieve a nontrivial two-qubit phase gate *U(T*) with 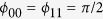 and 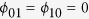, the maximal gate fidelity is achieved when the displacement amplitude is about 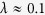 for almost all the values of *m*. The maximal gate fidelity is above 0.99 for both *m* = 2 and *m* = 3 with 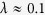. Here for the numerical simulation, we choose the system parameters as cavity frequencies *ω*_*a*_/2*π* = 10 GHz, *ω*_*b*_ = *ω*_*c*_ = *ω*_*a*_/*m*, the total evolution time *T* = 2*π*/*ω*_*b*_, and the resonator-resonator coupling strength *J* = 0.1|*ω*_*a*_ − *ω*_*b*_|.

### Errors and decoherence

**Figure 4:**
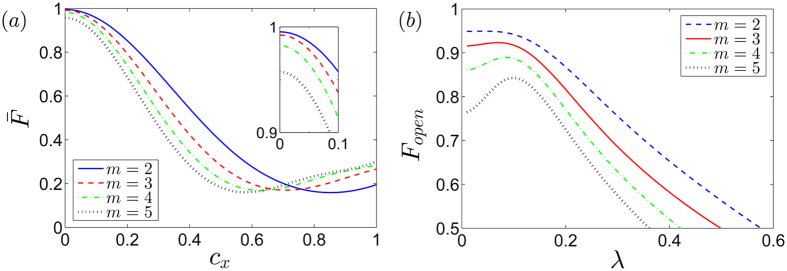
Gate performance in presence of imperfections. (**a**) Gate fidelity 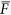 as a function of the nonzero transversal coupling strength *c*_*x*_. The inset is a zoom of the region of 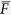 between 0.9 and 1. (**b**) State fidelity in the open system 
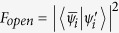
 with external noises 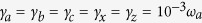, for the initial state prepared in the maximum entangled state 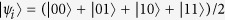. For the simulation, we choose the system parameters as cavity frequencies *ω*_*a*_/2*π* = 10 GHz, *ω*_*b*_ = *ω*_*c*_ = *ω*_*a*_/*m*, the total evolution *T* = 2*π*/*ω*_*b*_, and the resonator-resonator coupling strength *J* = 0.1|*ω*_*a*_ − *ω*_*b*_|.

We remark that all our simulations presented so far assume no loss in the system and it is inevitable that any realistic quantum system operates with imperfect controls and noisy environments. It is clear that our phase gate *U(T*) specifically requires the longitudinal coupling strength such that we have assumed that in the effective Hamiltonian Eq. (4) the transversal coupling coefficient is zero, i.e. *c*_*x*_ = 0. Although, in principle the coefficients 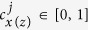 can be manipulated by the external fluxes, in realistic systems there might be some residual nonzero transversal couplings, i.e., 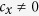, which will surely affect the performance of the two-qubit phase gate *U(T*). In Fig. 4(a), we show the numerical simulations of the two-qubit gate performance in the presence of the nonzero transversal couplings governed by Eq. (3). We note that for 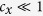, it doesn’t affect much of the gate performance. Moreover, for *c*_*x*_ < 0.6, smaller integers of *m* always gives better gate fidelity.

In additional to the errors induced by the imperfect control of the coupling strengths, our system is exposed to noisy environments. In circuit QED, decoherence arises from various environmental degrees of freedom and one of the key challenges is to minimize both the internal and the external noise sources. To better explore the performance of our gate proposal in realistic situations, dissipation induced by its coupling to the environment needs to be taken into account. However, the description offered by the standard quantum optical master equation breaks down in the ultrastrong coupling regime and open system analysis of the ultrastrongly-coupled systems needs to be carried out by studying dynamics of the microscopic master equation (see Methods). In Fig. 4(b), we show the performance of the two-qubit phase gate *U(T*) with *ϕ*_00_ = *ϕ*_11_ = *π*/2 and *ϕ*_01_ = *ϕ*_10_ = 0 for the initial state prepared in the maximally entangled state 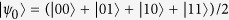 in presence of external noises. For any values of *λ* satisfying *λ* < 0.6, the fidelity in the open system case with the initial state prepared in the maximally entangled state is deceasing as *m* is getting larger. In this case, the maximal open-system fidelity can be archived is 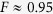 with *m* = 2 and 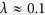.

## Discussion

To summarize, we have presented a scheme to realize ultrafast quantum computation in the ultrastrong coupling regime of a multi-resonator circuit QED system, where the flux qubits are ultrastrongly coupled to the middle TLR, and another two empty resonators are connected to the middle TLR through SQUIDs to assist the quantum gate operations. Numerical results show that the geometric phase gate in the system operates with a gate fidelity above 99% for certain choices of parameters. In addition to ultrafastness of the quantum gate operation at sub nanosecond time scale due to the ultrastrong coupling strength, our scheme offers another two advantages. (i) By resorting to the displacements of the intracavity fields of three resonators, the evolution of the system results in a nontrivial two-qubit geometric phase gate and thus it possesses global geometric features enabling noise-tolerant quantum computation against certain type noises. (ii) Depending on the amount of displacement on the resonator fields, the requirement of the coupling strength to realize a certain two-qubit phase gate can be greatly relaxed compared to the required coupling strength of 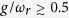 in ref. 25. By employing two empty resonators to contribute to the phase accumulation of the quantum phase gates, the requirement on the coupling strength can be further relaxed. Even more, the requirement on the coupling strength for a quantum phase gate can be multiply reduced if we generalize our scheme to a system consisting of N auxiliary transmission line resonators. The result is important for quantum informational and computational applications because our scheme based on multi-resonator circuit QED systems is as well applicable to other types of qubit-resoantor coupled systems, where ultrastrong coupling is not feasible. By using multiple resonators, our scheme can relax the requirement of strong coupling strength and hence speed-up quantum gate is possibly achievable even without ultrastrong coupling. Therefore, our scheme opens the possibility of implementing ultrafast quantum gates holding noise-resistant merits based on the advantages of geometric phases.

## Methods

Owing to the very high qubit-resonator coupling ratio *g*/*ω*_*a*_, the standard quantum optical master equation fails to describe the dynamics of ultrastrongly coupled systems. Open system analysis of an ultrastrongly coupled system can be carried out by studying dynamics of the microscopic master equation. In the following, we rewrite the system operators in the eigenbasis of the total system Hamiltonian, and by applying the standard Markov approximation and tracing out the reservoirs degrees of freedom, we arrive at the master equation in the presence of noises at very low temperature environment of 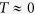
21,51 (generalization to 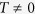 environments is straightforward),


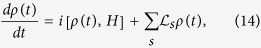


where the subscripts *s* stands for the qubit losses with *s* = *x, z* and for the cavity losses with *s* = *a, b, c*. The Liouvillian superoperator is defined as


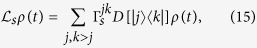


with |*j*〉 being the eigenstates of the total system Hamiltonian Eq. (1), 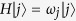, and the dissipator being


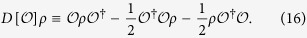


The relaxation coefficients are given by^21^,^51^


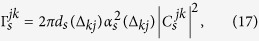


which depend on the system-bath coupling strength *α*_*s*_(Δ_*kj*_), the spectral density of the baths *d*_*s*_(Δ_*kj*_) at the respective transition frequency Δ_*kj*_ = *ω*_*k*_ − *ω*_*j*_, as well as on the transition matrix elements


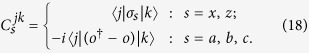


These relaxation coefficients can be interpreted as the full width at half maximum of each |*k*〉 → |*j*〉 transition. For simplicity, we assume *α*_*s*_(Δ_*kj*_) and *d*_*s*_(Δ_*kj*_) to be constant, and then the relaxation coefficients can be written in a compact form 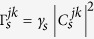, where *γ*_*s*_ are the standard damping rates of a weak coupling scenario. More specifically, *γ*_*x*_ and *γ*_*z*_ are the damping rates of the flux qubit associated with the transversal noise and longitudinal noise; *γ*_*a*_, *γ*_*b*_ and *γ*_*c*_ are the damping rates of the cavities.

## Additional Information

**How to cite this article:** Wang, Y. *et al*. Ultrafast quantum computation in ultrastrongly coupled circuit QED systems. *Sci. Rep.* **7**, 44251; doi: 10.1038/srep44251 (2017).

**Publisher's note:** Springer Nature remains neutral with regard to jurisdictional claims in published maps and institutional affiliations.
